# Ultrasound-Guided Continuous Transmuscular Quadratus Lumborum Block for Postoperative Analgesia in Patients Undergoing Radical Nephrectomy: A Randomized Controlled Trial

**DOI:** 10.7759/cureus.19120

**Published:** 2021-10-29

**Authors:** Shyam Prasad Mantha, Abhijit Nair, Praveen Kumar Kodisharapu, Poornachand Anne, Vibhavari M Naik, Basanth K Rayani

**Affiliations:** 1 Department of Anaesthesiology, Surgical Critical Care, Pain and Palliative Medicine, Basavatarakam Indo American Cancer Hospital and Research Institute, Hyderabad, IND; 2 Anesthesiology, Ibra Hospital, Ibra, OMN; 3 Department of Anaesthesiology, Surgical Critical Care, Pain and Palliative Medicine, Basavatarakam Indo-American Cancer Hospital and Research Institute, Hyderabad, IND

**Keywords:** ultrasound, regional anaesthesia, quadratus lumborum, nephrectomy, analgesia

## Abstract

Introduction

Ultrasound (US) guided transmuscular quadratus lumborum block (TMQLB) has been widely used as regional anaesthesia (RA) technique for managing postoperative pain after intraperitoneal and retroperitoneal procedures like nephrectomy, percutaneous nephrostomy, cholecystectomy, and also for hip surgeries. Although continuous epidural analgesia is considered the gold standard for most of these procedures, alternative techniques such as transversus abdominis plane (TAP) block and continuous rectus sheath block have also been used successfully. US-guided TMQLB seems to have more advantages than TAP block as it blocks the visceral afferents. With more cephalad spread of the local anaesthetic into the thoracic paravertebral space, it might block somatic pain from T6 to L2 as well.

Methods

After institutional ethics committee approval, we recruited 64 consecutive patients in the study and randomized them into two groups. Patients in the study group received bupivacaine (0.125%) and the control group received normal saline as a continuous infusion for 48 hours. Both groups were compared for 48 hours morphine consumption, time to first analgesic, hemodynamics, postoperative nausea/vomiting (PONV), sedation, and other adverse events.

Results

Data from 60 patients were analyzed. Forty-eight hours of morphine consumption in group A (study) was less than group B (7.4 ± 4.57 mg versus 11.86 ± 5.58 mg) and the time to first morphine requirement was 240 min (105-500) in group A compared to 90 min (90-225) in group B which was statistically significant. Demographic data, American Society of Anesthesiologists physical status, hemodynamics, Ramsay sedation score (RSS), and PONV were comparable in both groups.

Conclusion

Continuous US-guided TMQLB appears to be a safe and effective RA technique for managing postoperative pain after nephrectomy for up to 48 hours. Trial registration: German Clinical Trials Register-DRKS-ID: DRKS00014611.

## Introduction

The quadratus lumborum block (QLB) is an abdominal wall fascial block plane block described for providing perioperative analgesia for abdominal and lower limb surgeries [1,2]. There are four variants of the QLB described in the literature depending on the site of injection with respect to quadratus lumborum muscle (QLM). In an anterior or transmuscular QLB (TMQLB), the final needle position is anterior to QLM and the drug is deposited in the fascial plane between the psoas major muscle and QLM. In a posterior QLB, the needle's final position is posterior to the QLM and the drug is deposited in the fascia between the erector spinae muscle (ESM) and QLM. In posterolateral QLB, the final needle position is lateral to the QLM deeper to the transversus abdominis aponeurosis. In intramuscular QLB, the needle's final position is intramuscular, i.e., in the substance of QLM [3]. The initial description of TMQLB was by Børglum et al. in 2013 where the local anesthetic was injected anteriorly between the psoas major muscle (PMm) and the QLM [4].

The diffusion of the injected dye in the cadaver with TMQLB is cephalad between QLM and PMm from the lumbar site of injection. The main pathway is posterior to the arcuate ligaments and into the thoracic paravertebral space. The diffusion of the injected dye is reaching somatic nerves and the thoracic sympathetic trunk in the intercostal and paravertebral spaces. The lumbar plexus and lumbar sympathetic trunk were unaffected [5]. This was also demonstrated with computed tomography contrast studies in patients with QLB catheters in situ [6]. The results of US-guided QLB of cadavers conducted by Carline et al. confirmed Borglum et al. findings that TMQLB spread extends to nerve roots but is restricted to the lumbar region without the spread to the thoracic region as seen in dye injection in cadavers and magnetic resonance imaging (MRI) studies in volunteers using landmark technique [7]. We hypothesized that by performing an ipsilateral TMQLB in patients undergoing laparoscopic nephrectomy, postoperative morphine consumption will be lesser which was our primary outcome. The secondary outcomes were sedation scores, hemodynamics, and postoperative nausea and vomiting (PONV).

## Materials and methods

After obtaining Institutional Ethics Committee approval (IEC/2017/30 date February 26, 2017), we designed a double-blinded randomized controlled trial involving 60 patients undergoing elective laparoscopy-assisted nephrectomy under general anaesthesia. The trial was registered with German Clinical Trials Register-DRKS-ID: DRKS00014611. A written, informed consent was obtained from all patients after counseling them on how to report pain scores on a scale of 0-10. Patients with the American Society of Anesthesiologist’-physical status (ASA-PS) grades I and II undergoing radical nephrectomy with unilateral abdominal incision were included in the study. The duration of recruitment was from March 2017 to August 2020. Patients unable or unwilling to give informed consent, those with a history of relevant drug allergy, patients currently using other analgesics preoperatively or who had current acute or chronic pain or were receiving medical therapies considered to result in tolerance to opioid, deranged coagulation profile, severe hepatic, renal dysfunction, and infection at the block site were excluded. Patients who required a midline abdominal incision were also excluded as it would require a bilateral QLB.

Randomization was done on the basis of computer-generated randomization codes. Patients were allotted study numbers on the day of surgery after fulfilling all the inclusion criteria. Informed consent was obtained from all the patients. A senior anesthesiologist trained in US-guided regional anaesthesia (RA) techniques with more than five years of experience performed all the blocks. The assessing anesthesiologist and the patient were blinded but the performing anesthesiologist was not blinded.

After confirming six hours nil by mouth status, patients were shifted to the operation room and an appropriately sized intravenous (IV) access was secured and essential monitoring was established (non-invasive blood pressure, pulse oximeter, electrocardiography: leads II, V5). IV midazolam 0.03 mg/kg was used as pre medication. General anaesthesia was induced with IV propofol 2-2.5 mg/kg and fentanyl 2 μg/kg. Neuromuscular blockade was achieved with IV atracurium 0.5 mg/kg and tracheal intubation was performed with an appropriately sized endotracheal tube. Anaesthesia was maintained on volume-controlled ventilation with oxygen-medical air (1 L fresh gas flow on circle absorber) with isoflurane as maintenance anesthetic with dial concentration titrated to achieve a minimum alveolar concentration of 1. Intraoperatively patients were administered additional 1 μg/kg IV fentanyl if heart rate and blood pressure increased by more than 20% from baseline and IV paracetamol 1 g towards skin closure which was continued 8th hourly in the postoperative period. Isoflurane along with Air/O_2_ mixture is used for the maintenance of anaesthesia. Dexamethasone 8 mg IV is given at the end of the procedure as antiemetic. There was no port-site infiltration of LA done by surgeons after retrieving the specimen.

**Figure 1:**
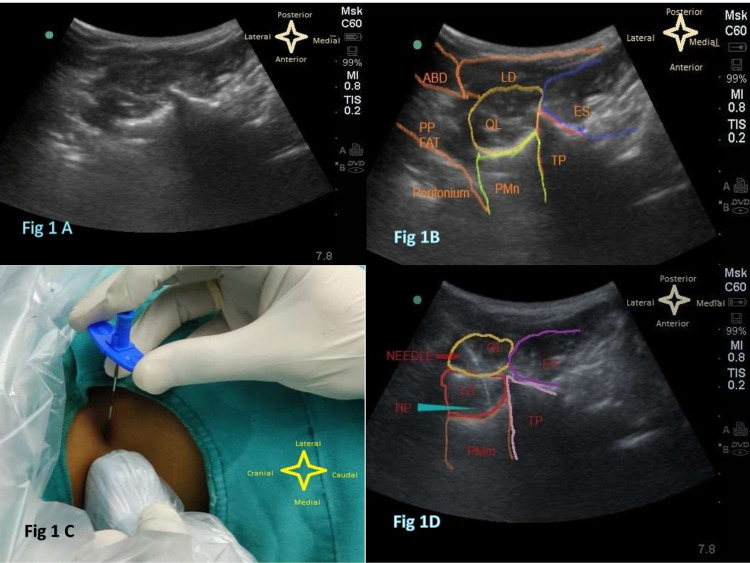
Ultrasound image showing the needle path and tip with relevant sonoanatomy The figure is also showing quadratus lumborum muscle (QLM), erector spinae muscle (ES), transverse process (TP), psoas major muscle (PMn), latissimus dorsi muscle (LD), abdominal muscles (ABD), pre-peritoneal fat (PP FAT)

**Figure 2:**
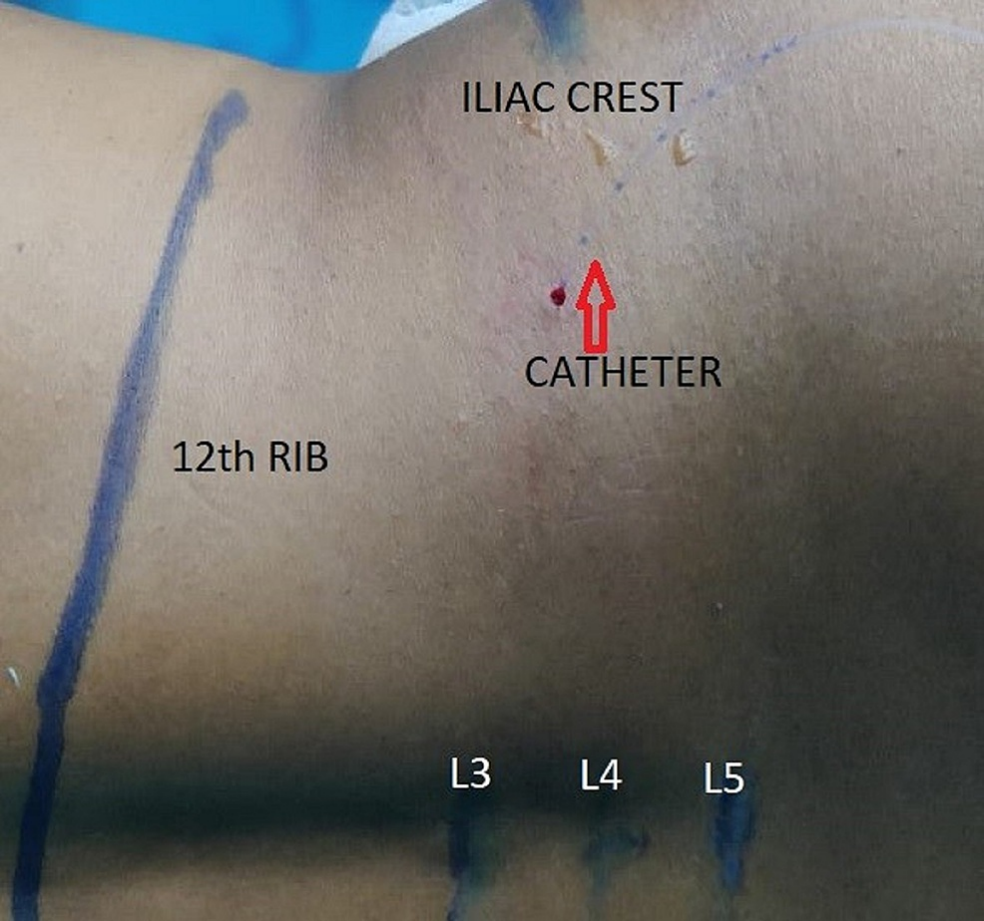
Image showing the final position of the catheter placement with marking of 12th rib, iliac crest, and lumbar spine 3,4,5

At the end of the surgery, the patient was placed in a lateral position with the operated side upwards. Under the aseptic technique, a curvilinear probe (6 to 4 MHz; Sonosite Inc., USA) was placed transversely cranial to the iliac crest and the probe was slided towards the spine dorsally without changing the transverse probe orientation. The transverse process of L4 and QLM was identified which is inserted into the tip of the transverse process. The PMm was identified anterior and the ESM posteromedial to QLM forming the cloverleaf pattern of shamrock (Figure 1A-1B). A 16 G Tuohy needle (Portex, Smiths Medical) was inserted in-plane technique from the lateral side of the curvilinear transducer and was advanced trans muscularly through QLM to penetrate the ventral fascia of the muscle (Figure 1C). The needle direction was anteromedial with the tip of the needle close to the tip of the transverse process of L4 vertebrae (Figure 1D). Hydro-dissection of the plane between QLM and PMm was done and an 18G epidural catheter (Portex, Smiths Medical) was inserted through the Tuohy needle with the Huber tip facing cranially and 5 cm of the catheter was kept in the plane created for continuous infusion in the postoperative period (Figure 2 and Video 1). The performing anesthesiologist injected a loading dose of 20 ml of the study drug (as per randomization). Group A patients received 0.125% of bupivacaine 20 ml as loading dose and group B received placebo, i.e., 20 ml normal saline by above-mentioned approaches group A patients received 0.125% of bupivacaine at the rate of 8 ml/hour as a continuous infusion via the quadratus lumborum catheter for 48 hours. Group B received 0.9% of normal saline infusion at the rate of 8 ml/hour for 48 hours. The infusion syringes used in the postoperative period were also labeled with the study number. The catheter was removed after 48 hours with an adequate time interval gap from the last low molecular weight heparin injection as per ASRA guidelines. After removal of the catheter, the site and lumbar region were checked for any swelling, hematoma.

**Video 1:** The video demonstrates ultrasound-guided transmuscular quadratus lumborum block with catheter insertion for continuous analgesia

Visual analogue scale (VAS) was used to assess pain postoperatively on a scale of 10 cm where 0 was no pain and 10 cm was the worst unbearable pain. IV paracetamol 1 g or 20 mg/kg in patients less than 50 kg was given in the postoperative ward every sixth hour. IV morphine was used as rescue analgesia if VAS score more than 4/10 (2 mg IV for patients less than 50 kg and 3 mg IV for patients more than 50 kg) reassessment was done 30 minutes after giving rescue analgesia. VAS scores, Ramsay sedation score (RSS), PONV, and total morphine consumption at 1, 6, 12, 24, 36, and 48 hours post-surgery and recorded in a high dependency unit which was monitored and documented by trained nursing staff in the high dependency unit.

The sample size was decided based on a pilot study. In our pilot study which comprised 12 patients, that is, 6 in each group, the mean morphine consumption in 48 hours and SD in the study group was 6.33 ± 2.06 mg and in the control group was 9 ± 6.29 mg. Based on this information, for a type-1 error of 0.05 and for a power of 0.8, the sample size required was 56 patients, that is, 28 patients in each group. To avoid possible drops out, loss of data, we decided to recruit 32 patients in each group.

Data were collected and entered into a Microsoft Excel sheet for analysis. Data were expressed as mean ± standard deviation or median-interquartile range as appropriate. The Kolmogorov-Smirnov test was used to determine whether the variables were normally distributed. Unpaired t-test or Mann-Whitney U test was used for analysis for continuous data. The chi-square (χ^2^) test was used to compare qualitative variables. Statistical analysis was performed using GraphPad Prism 5 for Windows (GraphPad Software, La Jolla, CA, USA). A P-value <0.05 was considered statistically significant.

## Results

**Figure 3:**
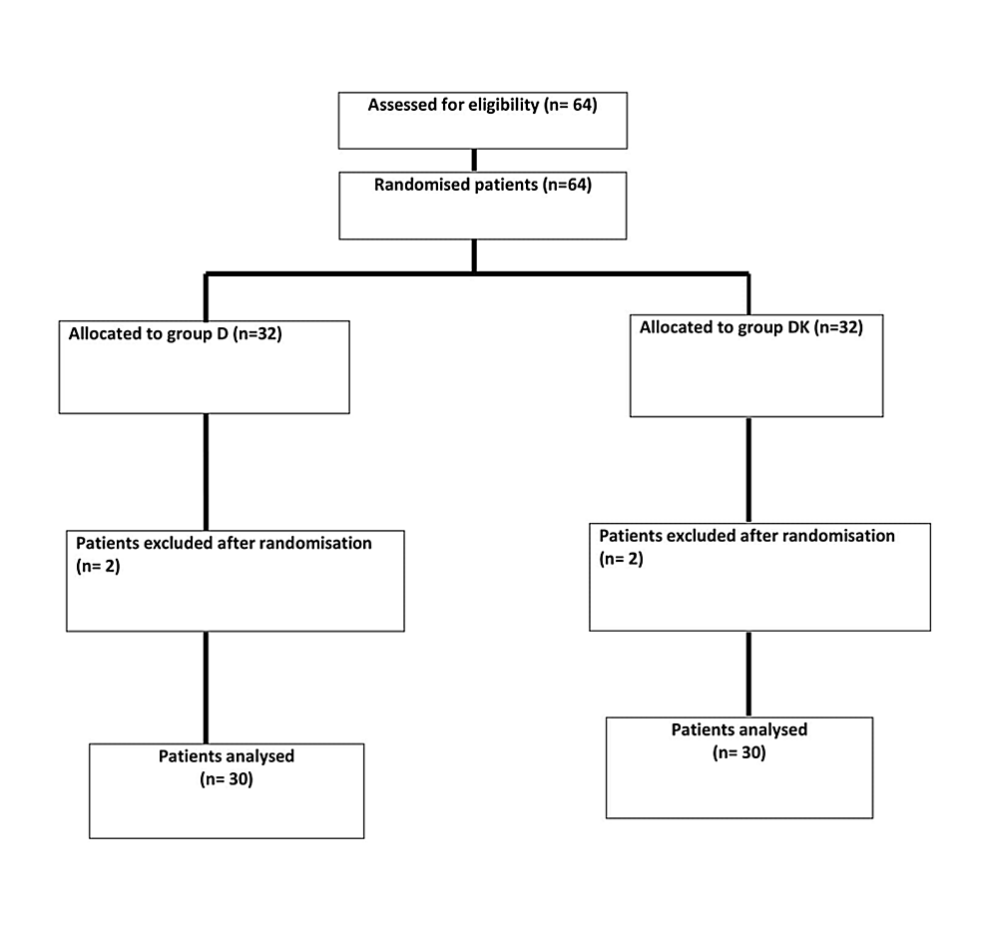
CONSORT (Consolidated Standards of Reporting Trials) flow diagram

We recruited 64 patients, 32 patients in each group. The Consolidated Standards of Reporting Trials (CONSORT) flow diagram is depicted in Figure 3.

**Figure 4:**
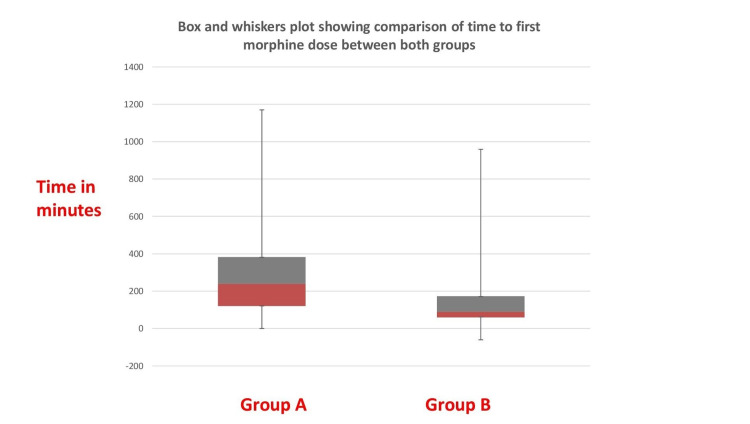
Box and whiskers plot showing the comparison of time to first morphine dose between both groups

The four patients (n =4) were excluded from the study (n=2) due to midline incision (n=2) and postoperative delirium (n=2). Demographic data like age, weight, gender, BMI, and ASA-PS were comparable in both groups. PONV was comparable in both groups (Table 1). Forty-eight hours morphine consumption was 7.4 + 4.57 mg in the study group and in the control group was 11.86 + 5.58 mg which was statistically significant (p=0.002). This was our primary outcome. The median time to first morphine dose was 240 minutes (105-500) in the study group compared to 90 minutes (57-225) in the control group which was statistically significant (p=0.034; Figure 4).

**Table 1:** Comparison of demography, duration of surgery, intra-operative fentanyl used, first morphine dose with 24 hours morphine consumption, and PONV score in both groups ASA: American Society of Anesthesiologists physical status, PONV: postoperative nausea and vomiting, NS: not significant, S: significant

Variable	Group A (n= 30)	Group B (n=30)	P-value
Age	50.26+10.39	53.23+8.36	0.114 (NS)
Weight (kg)	61.73+11.84	63.3+13.21	0.315 (NS)
ASA (I/II/III)	3/25/2	6/24/0	0.22(NS)
Gender (M/F)	17/13	18/12	0.793 (NS)
First morphine dose (min)	240 (105–500)	90 (57–225)	0.034 (S)
PONV score	13 (43.33%)	19 (63.33%)	0.12 (NS)
48 hours morphine consumption (mg)	7.4+4.57	11.86+5.58	0.002 (S)

There was no statistically significant difference in heart rate, systolic blood pressure (SBP), diastolic blood pressure, and mean arterial pressure at 0, 1, 3, 6, 12, 24, 36, and 48 hours in both groups except SBP at 48 hours (Table 2).

**Table 2:** Comparison of baseline parameters: heart rate, SBP, DBP, MAP at various intervals over 48 hours HR: heart rate, SBP: systolic blood pressure, DBP: diastolic blood pressure, MAP: mean arterial pressure *Unpaired T-test

Variable		Group A (n=30) mean + SD	Group B (n=30) mean + SD	P-value
0 hr	HR	83.3±17.63	80.97±16.98	0.603
	SBP	133.07±16.43	134.17±15.99	0.7936
	DBP	77.4±10.02	78.53±9.72	0.6582
	MAP	96.17±10.25	96.17±11.13	1
1 hr	HR	84.2±16.17	81.95±16.56	0.593
	SBP	138.57±17.07	134.13±13.44	0.2683
	DBP	77.4±9.11	79.43±8.04	0.3631
	MAP	97.77±9.2	97.77±7.75	1
3 hr	HR	80.17±18.09	83.97±16.7	0.4013
	SBP	134.07±13.51	134.73±12.09	0.8411
	DBP	78.07±9.89	79.63±9.26	0.5288
	MAP	96.37±9.6	97.9±8.9	0.5239
6 hr	HR	85±12.75	86.4±14.37	0.6913
	SBP	131.93±11.88	131.4±15.64	0.882
	DBP	77.6±11.16	80.77±9.24	0.236
	MAP	95.73±10.02	97.63±9.54	0.455
12 hr	HR	87.3±16	90.87±15.41	0.382
	SBP	127.03±9.62	128.6±12.22	0.583
	DBP	77.53±9.13	75.07±9.16	0.30
	MAP	93.9±7.53	92.97±8.59	0.656
24 hr	HR	87.97±10.49	92±13.16	0.194
	SBP	129.67±13.68	131.97±12.95	0.506
	DBP	76.17±9.46	78.93±10.15	0.279
	MAP	94±9.49	96.53±10.12	0.321
36 hr	HR	87.27±11.49	90.1±11.92	0.352
	SBP	130.33±13.25	130.43±12.37	0.976
	DBP	77.97±11.03	79.63±7.69	0.499
	MAP	95.37±9.82	96.57±7.96	0.605
48 hr	HR	91.5±11.2	92.33±9.63	0.758
	SBP	126.33±12.5	133.7±13.42	0.031
	DBP	77.4±10.91	80.8±9.11	0.195
	MAP	93.67±10.3	98.3±8.65	0.0643

**Figure 5:**
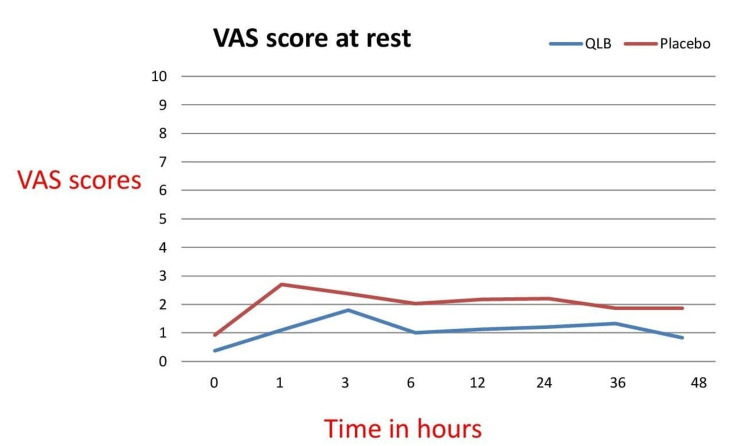
Line graph diagram of the VAS scores of both groups at rest VAS: visual analogue scale

VAS score was documented at 0, 1, 3, 6, 12, 24, 36, and 48 hours both at rest and on movement. VAS scores at rest were significantly better in a study group at 1, 6, 12, 24, and 48 hours (Table 3 and Figure 5).

**Table 3:** Comparison of VAS scores at rest and movement over 48 hours VAS: visual analogue scale *Unpaired T-test

VAS (hour)		Group A (n=30)	Group B (n=30)	p-Value*
0	Rest	0.37	0.93	0.089
	Movement	0.47	1	0.127
1	Rest	1.1	2.7	0.004
	Movement	1.5	3	0.011
3	Rest	1.8	2.37	0.118
	Movement	2.2	2.97	0.06
6	Rest	1	2.03	0.003
	Movement	1.77	2.47	0.042
12	Rest	1.13	2.17	0.005
	Movement	2	2.83	0.025
24	Rest	1.2	2.2	0.005
	Movement	2.2	2.77	0.097
36	Rest	1.33	1.87	0.064
	Movement	2	2.7	0.049
48	Rest	0.83	1.87	0.002
	Movement	1.7	2.67	0.004

**Figure 6:**
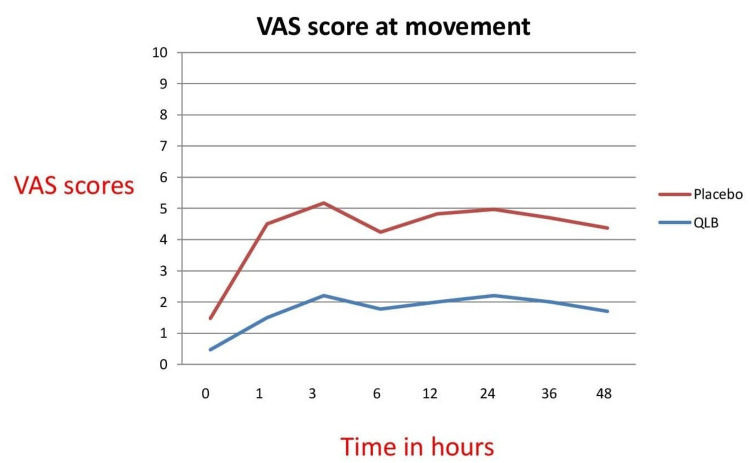
Line graph diagram of the VAS scores of both groups on movement VAS: visual analogue scale

VAS scores at movement were significantly better at 1, 6, 12, 36, and 48 hours in the study group (Figure 6). RSS was more in the study group at 0, 1, 3 hours when compared to the control group which could be explained because they received local anaesthetic (LA) which provided pain relief, unlike saline which was injected in the control group. However, after three hours, RSS was comparable in both groups (Table 4). There was no statistically significant difference in postoperative nausea vomiting scores in both groups.

**Table 4:** Comparison of Ramsay sedation scores between two groups

RSS	Group A (n=30)	Group B (n=30)	p-Value*
0 hr	5.13±0.86	4.43±0.67	0.009
1 hr	3.66±1.37	2.83±0.98	0.0091
3 hr	2.7±0.83	2.26±0.69	0.0294
6 hr	2.4±0.56	2.16±1.78	0.484
12 hr	2.2±0.48	2.06±0.52	0.283
24 hr	2.23±0.43	2.13±0.57	0.446
36 hr	2.05±0.25	2.03±0.61	0.868
48 hr	2.1±0.3	2.06±0.52	0.716

## Discussion

In this single-center, randomized, double-blinded, controlled trial comparing postoperative pain relief and 48 hours morphine consumption after laparoscopic nephrectomy, we demonstrated that a continuous QLB with 0.125% bupivacaine infusion produced clinically significant analgesia in the first 48 hours when compared to saline infusion.

To the best of our knowledge, this is the first randomized controlled study in which an anterior QLB study was used in laparoscopy-assisted nephrectomy cases for postoperative analgesia for 48 hours with an indwelling catheter for continuous infusion. In a study by Zhu et al., the authors recruited 60 patients in two groups: the QLB group and saline as a placebo [8]. Although otherwise similar to our study, Zhu et al. performed single shot subcostal QLB. The result of the study indicates that 48-hour morphine consumption was significantly less in the study group compared to the control group. The median time of the first dose of morphine was more than 120 minutes and statistically significant with better VAS scores at most of the times in 48 hours. In a study by Dam et al., the authors randomized 60 patients undergoing nephrolithotomy into two groups, one group received TMQLB (unilateral 30 ml LA), and another received saline (a volume of 30 ml) [9]. On analysis, authors concluded that after TMQLB there was lesser postoperative opioid consumption and shorter length of hospital stay. In another study by Dam et al., authors randomized 50 patients in two groups scheduled to undergo robotic and laparoscopic nephrectomy [10]. The intervention group received bilateral US-guided TMQLB with 30 ml of 0.375% ropivacaine on each side. The control group received a similar volume of saline in the same plane. On analysis, authors concluded that oral morphine equivalent consumption was significantly lower in the QLB group than placebo at 12 hours with significantly prolonged time to the first opioid in the QLB group. In this study, bilateral single-shot blocks were performed preoperatively using a very large volume of LA (60 ml). However, the authors did not compare the intraoperative remifentanil consumption even after preoperative blocks. All three studies used single-shot QLB. In our study, QLB was unilateral and moreover continuous using an indwelling catheter which possibly provided a better and longer duration of postoperative analgesia.

Kadam et al. randomized 82 patients into two groups [11]. The first group received bilateral TMQLB postoperatively followed by continuous LA infusion for 48 hours. Other groups received continuous LA infusion via pre-peritoneal catheters placed postoperatively by the surgeon (pre-peritoneal, sub-fascial, and subcutaneous plane). Initially, 60 ml of LA (20 ml in each plane) was injected followed by continuous infusion for 48 hours. On analysis, authors concluded that although pain scores were better in the QLB group the overall fentanyl consumption was similar with preperitoneal catheter infusion, more cost-effective than QLB. Aditianingsih et al. randomized 62 patients undergoing laparoscopic donor nephrectomy patients to receive bilateral TMQLB in one group (up to 25 ml on each group) and continuous epidural anaesthesia in another group (0.25% bupivacaine at 6 ml/hour) [12]. On analysis, authors found that 24 hours morphine consumption, time to first rescue analgesia, PONV was comparable between QLB and epidural group. In our study, we performed ipsilateral blocks at the end of surgery. Moreover, our patients received continuous LA infusion for 48 hours. This could be the reason for better pain scores and lesser morphine consumption in our patients who received the intervention.

Pain in laparoscopic-assisted radical nephrectomy is multifactorial. It could be due to port site pain, incision, visceral pain, shoulder tip pain due to pneumoperitoneum. In QLB, LA spread possibly covers T5 to L1 dermatomes. Several studies have shown that QLB has a good visceral pain blockade compared to the transversus abdominis plane (TAP) as the block is more posterior [13]. Due to the wide dermatomal cover with QLB, incisional pain is covered. QLB is a US-guided fascial plane block of the posterior abdominal wall. TMQLB is considered technically difficult to perform as the needle traverses the QLM anteriorly. There is a risk of injuring the lumbar vessel which traverses horizontally across fascia planes. The close proximity to the preperitoneal fascia and presence of the kidney are the challenges to deal with.

In the technique described by Borglum et al., a curvilinear probe is used with the entry of the needle anteromedially which decreases the chances of needle entry into pre-peritoneal fat and also reduces the chance of injury to the kidney [14]. In the earlier description, needle entry was through the erector spinae muscle before reaching the fascial plane. This block should be performed at the level of the transverse process using color doppler as well so as to avoid injury to vessels which could be present in the needle path or in the myofascial plane.

In our study, one patient has quadriceps weakness post-operatively. This could be because of the loading dose of 20 ml which recovered in a few hours. On maintenance infusion at 8 ml/hour, there was no weakness noticed for the next 48 hours. There were no other procedure-related complications. In the postoperative period, a regular check of the site of injection was done to look for any swelling and tenderness. The catheter was removed after 48 hours as per American Society of Regional Anaesthesia (ASRA) guidelines for low molecular weight heparin [14]. The site of injection was noted for any signs of local infection. Complications like bleeding, infection related to the procedure might occur. severe hypotension was reported in two cases 30 mins after a block was documented [15]. In a retrospective study conducted by Ueshima and Hiroshi, the incidence of quadriceps muscle weakness was reported highest in TMQLB as compared to other types of QL block [16]. Wikner reported unexpected sensory loss at L2 dermatome and weakness of psoas, iliacus, and quadriceps muscle for 18 hours [17]. Motor block after QLB is possible because LA seeps into lumbar plexus elements situated in PMm. This is possible if LA is not deposited in the fascial plane between QLM and PMm and accidentally gets deposited in PMm.

This study had several limitations. The block was not offered to patients with BMI more than 35 kg/m^2^ and in patients with a renal impairment which may be present in patients with renal cell carcinoma. We used 20 ml LA as a bolus and an infusion at 8 ml/hour for 48 hours, i.e., 384 ml. We did not estimate serum bupivacaine levels although none of the patients showed any clinical signs of LA toxicity. We suggest estimation of serum LA concentration in future studies if the continuous infusion is used after a QLB. The dermatome innervation was not assessed.

## Conclusions

This prospective, single-center, randomized controlled trial investigated the efficacy of US-guided, ipsilateral anterior TMQLB in patients undergoing laparoscopic nephrectomy. The results revealed that the intervention provided statistically significant, opioid-sparing analgesia in patients who received the block than a placebo. Thus, we conclude that US-guided, unilateral, anterior TMQLB is a safe and effective US-guided regional anesthesia technique that provides opioid-sparing analgesia for laparoscopic nephrectomy. We suggest a continuous LA infusion through an indwelling catheter in the myofascial plane for sustained postoperative analgesia.
